# Using a Candidate Gene-Based Genetic Linkage Map to Identify QTL for Winter Survival in Perennial Ryegrass

**DOI:** 10.1371/journal.pone.0152004

**Published:** 2016-03-24

**Authors:** Cristiana Paina, Stephen L. Byrne, Bruno Studer, Odd Arne Rognli, Torben Asp

**Affiliations:** 1 Department of Molecular Biology and Genetics, Science and Technology, Aarhus University, Slagelse, Denmark; 2 Institute of Agricultural Sciences, ETH Zürich, Zürich, Switzerland; 3 Department of Plant and Environmental Sciences, Norwegian University of Life Sciences, Ås, Norway; Aberystwyth University, UNITED KINGDOM

## Abstract

Important agronomical traits in perennial ryegrass (*Lolium perenne*) breeding programs such as winter survival and heading date, are quantitative traits that are generally controlled by multiple loci. Individually, these loci have relatively small effects. The aim of this study was to develop a candidate gene based Illumina GoldenGate 1,536-plex assay, containing single nucleotide polymorphism markers designed from transcripts involved in response to cold acclimation, vernalization, and induction of flowering. The assay was used to genotype a mapping population that we have also phenotyped for winter survival to complement the heading date trait previously mapped in this population. A positive correlation was observed between strong vernalization requirement and winter survival, and some QTL for winter survival and heading date overlapped on the genetic map. Candidate genes were located in clusters along the genetic map, some of which co-localized with QTL for winter survival and heading date. These clusters of candidate genes may be used in candidate gene based association studies to identify alleles associated with winter survival and heading date.

## Introduction

In order to flower, perennial ryegrass (*Lolium perenne*) requires a period of primary induction, consisting of low temperature (vernalization) and short photoperiods, followed by a period of secondary induction, consisting of longer photoperiods [1]. Under vernalization, plants adjust to low temperatures and, at the same time, begin their transition to reproductive phase. Connections between the genetic components of cold response and vernalization have already been observed in temperate cereals, where the expression levels of the *VERNALIZATION 1* gene (*VRN1*) correlates with the expression of cold response regulators like the *C-repeat binding factor* (*CBF*), *cold regulated* (*COR*) and *late embryogenesis abundant proteins* (*LEA*) [2–4]. Furthermore, allelic variation at *VRN1* in wheat is associated with differences in freezing tolerance [2]. In barley, it has been suggested that a common signaling pathway may be involved in initiating both cold acclimation and the vernalization response [5]. Recently it was shown that barley *CBF2*, *CBF4*, and *CBF9* genes contain *VRN1*-binding sites, suggesting direct interaction between *VRN1* and these low temperature induced genes [6].

Vernalization response and winter survival are quantitative traits controlled by multiple loci with relatively small individual effects. Quantitative Trait Loci (QTL) for heading date were previously reported on all the seven linkage groups (LG) of perennial ryegrass [7–15], and the putative genes underlying two major QTL have already been proposed [8, 16]. QTL related to winter hardiness have also been reported in perennial ryegrass on LG 2, 4, 5, 6 and 7 [13, 17–19]. All the above QTL studies used freezing tolerance as a measure of winter hardiness. However, forage grass breeders prefer field testing in order to assess winter survival (WS) [20]. Furthermore, it has been shown in alfalfa that field measurements of WS reflect the winter hardiness of a plant much better than freezing tolerance [21]. WS QTL were previously detected on LG 2, 4, 6, and 7 in a perennial ryegrass population, where the QTL on LG 2 and 4 located close to QTL for high molecular weight fructan content [22]. In another study, Yamada et al. detected one freezing tolerance QTL on LG4 with no significant correlation to heading date, and no WS QTL [13]. In an interspecific hybrid ryegrass population developed from a cross between an annual and a perennial genotype, WS QTL were reported on LG 1, 2, 3, 4, and 5, where some of these QTL co-localized with QTL for freezing tolerance and/or fall growth, with significant correlation between these traits [23]. A study in meadow fescue identified WS QTL on LG 1, 2, 5, and 6, and frost tolerance QTL on LG 4, 5, 6, and 7 [24].

The VrnA population used in this study originates from a cross between the Danish ecotype Falster with a strong vernalization requirement, and the genotype Veyo from an Italian cultivar with no vernalization requirement [10]. QTL for seed yield and fertility traits, including vernalization response and heading, have previously been identified in this population [10, 11]. The present study aims to genetically map candidate genes for vernalization and cold response and to identify genes that co-locate with QTL for winter hardiness and with previously identified QTL for heading date. The specific aims were (i) to design an Illumina GoldenGate 1,536-plex candidate gene based oligo-pool genotyping assay, (ii) to construct a high confidence candidate gene based genetic linkage map, (iii) to identify new QTL for WS, (iv) to relate these mapped candidate genes to previously identified QTL for heading date and vernalization response, and (v) to anchor scaffolds of the perennial ryegrass genome [25] using the genetic map.

## Material and Methods

### SNP discovery

The SNP discovery was based on a set of transcripts previously shown to have expression level changes at various time points under vernalization/cold treatment, including: (1) a temperature drop to 6°C after eight weeks under 15°C and eight hours photoperiod, (2) throughout nine weeks of vernalization/cold treatment at 6°C and eight hours photoperiod, and (3) the shift to long day conditions with 20°C and 16 hours photoperiod. These transcripts and their expression patterns observed during the treatment were previously described [26] and are publicly available in the EMBL-EBI ArrayExpress repository, experiment accession E-MTAB-2623. Sequences related to temperature and light induced stress response are present in the data set, as well as genes related to vernalization and induction of flowering. This set of transcripts was considered to contain good candidates for the induction of flowering and cold response. The transcripts originate from the two perennial ryegrass genotypes Falster and Veyo with contrasting vernalization requirement. In addition, a number of sequences corresponding to genes involved in fructan metabolism and the *VERNALIZATION INSENSITIVE-LIKE* (*VIL*) genes present in the transcriptome of the two genotypes were also included. In order to identify SNP polymorphisms, Illumina RNA-Seq reads generated from one of the parents of the VrnA population named F1-30 [27], offspring of the Falster and Veyo genotypes, were aligned against the candidate gene transcripts originating from Falster and Veyo. Heterozygous loci (SNPs) observed in the F1-30 genotype were expected to segregate within the mapping population. Bowtie [28] was used for the alignment, allowing a maximum of two mismatches (-v 2). Variants were called using the *mpileup* function from Samtools v1.7 [29]. SNPs were identified according to several criteria detailed in the S1 File.

### SNP validation

A subset of 24 SNPs was selected for validation by High Resolution Melting (HRM) analysis. These SNPs were chosen to cover the whole range of selected variant frequencies (25–75%) and to have a wide range of read coverage, with between seven and up to 4,006 reads aligned at the SNP position (S2 File). The parents and twelve plants from the VrnA mapping population were screened using HRM analysis following a previously described protocol [30].

### Illumina GoldenGate assay design and genotyping

After removing SNPs corresponding to sequences already mapped in the VrnA population [31], a total of 5,878 SNPs were considered for GoldenGate assay design using the Illumina Assay Design Tool (ADT). A final set of 1,536 SNPs was selected based on their ADT quality scores for Illumina GoldenGate genotyping using a custom made oligo pool assay. The final set included 933 SNPs derived from Falster and 603 from Veyo transcripts. A total of 16 SNPs were included in duplicates, to be used as a control parameter for genotyping and map construction. For all the other SNPs, special attention was paid to select only one SNP per differentially expressed transcript. The lowest Illumina SNP scores selected were 0.56 for Falster and 0.589 for Veyo derived SNPs. The genotyping was performed as a custom service by AROS Applied Biotechnology A/S Denmark, according to the Illumina protocol. One hundred and sixty-four individuals of the VrnA population were genotyped, with the parental genotypes analyzed in duplicates. The resulting genotyping data was manually inspected and corrected using the Illumina Genome Studio software v2011.1. The sequences with the SNP positions marked, as submitted for the genotyping array, are presented in the S3 File.

### Linkage map construction

The genetic linkage map was constructed in two steps using the JoinMap 4.1 software [32]. In the first step, a high confidence map was constructed using only the fully informative SNPs that showed an F2-type segregation pattern (homozygous in both grandparents, heterozygous in both parents (F1), and segregating in the VrnA population (F2). From here on, this map will be referred to as the F2-type map. The marker order from this map was further used as fixed order to build the final map, including all the available SNPs. A total of 57 markers from the previously developed perennial ryegrass linkage map [31], selected to span all seven LG, were also included in the final map. This enabled the maps to be linked. Markers were assigned to LG based on independence LOD scores and strongest cross link values. The Maximum Likelihood (ML) algorithm was used for map construction. The nearest neighbor fit, the nearest neighbor stress, the plausible positions, and the map length were used as control parameters. Once the optimal order within the F2-map was established, the remaining markers were added. Markers were placed into ‘bins’ when the order of markers could not be ascertained with sufficient confidence. This is to be expected when mapping large numbers of markers in an experimental cross of limited size.

The most plausible positions of each marker in the final map were used to estimate the density of the candidate genes on the linkage map.

### Phenotypic characterization for winter survival and heading date

Previous studies have highlighted significant interaction between genotype and environment in case of winter survival [33]. A higher latitude was deliberately chosen to evaluate the plant material in order to get a better estimate of winter survival.

Clonal ramets of the two parents and the VrnA mapping population were established in a field experiment at the Norwegian University of Life Sciences, Ås (59°40’N, 10°48’E), located in the southern part of Norway, in spring 2005 using a randomized complete block design with three replicates. The tillers were selected as the same developmental stage. WS was visually scored on the 2^nd^ of May 2006 using a scale from 0–10, where 0 corresponds to plants that did not survive the winter and 10 corresponds to plants having no visible injuries after winter. Three different methods (LSD, Tukey, REGWQ) were used to test for significant differences between the genotypes of the mapping population, and between the parents and grandparents, using PROC GLM in SAS (SAS Institute Inc., Cary, NC, USA).

A number of plants did not survive winter, others prooved a poor survival, and due to this, the phenotyping experiment could unfortunately not be repeated in the next year.

Previously reported phenotypes for heading date expressed as growing degree-days to heading of the VrnA population were used for this study. This data was recorded in two consecutive years, 2004 and 2005 [11].

Spearman’s rank correlation coefficient was employed in R [34] to test for correlation between winter survival and heading date.

### QTL analysis

Out of 164 VrnA genotyped individuals, three showed many more crossing-over events than average and were eliminated from further analysis. The QTL analysis was based on the most informative SNPs, represented in the high confidence F2-type map. The *qtl* R package [35] was used for QTL mapping in R [34], with the Multiple QTL Mapping (MQM) algorithm [36] and the multiple imputations model with 500 imputations (*n*.*draws = 500*, *step = 1*, *err = 0*.*001*). Automatic cofactor selection was performed using two different approaches. In the first modeling, the starting point for cofactor selection was a set of 146 randomly selected markers (*mqm_auto*), and after performing several iterations a final set of cofactors was established. In the second modeling, the starting point for cofactor selection was a set consisting of every fifth marker (*mqm_backw*). To establish a genome wide LOD threshold, 1000 permutations were performed and the LOD value at a 5% significance level was recorded as a threshold above which a QTL was considered to be significant. Each data set was queried for the presence of QTL. QTL above and close to the established genome wide LOD threshold were tested in the model. The MQM mapping steps were repeated until the QTL remained stable and no other significant QTL were detected. Both cofactor selection methods eventually converged on the same model. The 1.5 LOD support interval and the approximate 95% Bayesian credible interval were calculated for each detected QTL. The number of candidate genes reported to map in the confidence interval of a QTL refers to the overlap interval of the two estimation methods. MapChart version 2.2 [37] was used to create the final charts.

### Genome scaffold anchoring

Marker sequences from the Illumina GoldenGate assay (as presented in the S3 File) and from the previously developed transcriptome map [31] were aligned to the perennial ryegrass genome [25] using BLAT [38]. Only sequences that aligned to a single genomic location were retained. These were then used as markers to integrate the information from both genetic linkage maps for ordering scaffolds from the genome assembly. This was done using ALLMAPS [39] and equal weighting was applied to both maps. The chain file with the anchored scaffolds is presented in S4 File.

## Results and Discussion

### GoldenGate genotyping assay and the linkage map

A total of 45,064 SNPs were identified using RNA-Seq reads from one parent of the VrnA population and the sequences of the candidate genes as reference. After applying filtering steps, this set was reduced to 5,985 high quality SNPs within a total of 2,494 candidate genes (S1 File).

Out of the 24 SNPs selected for the HRM validation, one did not show amplification under the tested conditions, one was monomorphic, and three indicated the presence of paralogous genes. The remaining 19 SNPs (79%) were confirmed. These covered the whole selected range of variant frequencies and a wide range regarding the number of reads covering the SNP (7–3,101), as detailed in the S2 File.

The final set of 1,536 SNPs corresponding to transcripts showing differential expression during vernalization and the transition to long days was selected based on their Illumina scores and are represented on the genotyping array. Out of these, a total of 151 SNPs (9.83%) were further discarded due to low signal intensity on the array or because they formed unclear clusters, and 115 SNPs (7.48%) proved monomorphic. Another 110 SNPs (7.16%) showed severe segregation distortion, suspect linkages or high stress within the linkage groups and were excluded from further analysis. The remaining 1,160 SNPs (75.52%) were used for constructing the genetic linkage map.

**Fig 1:**
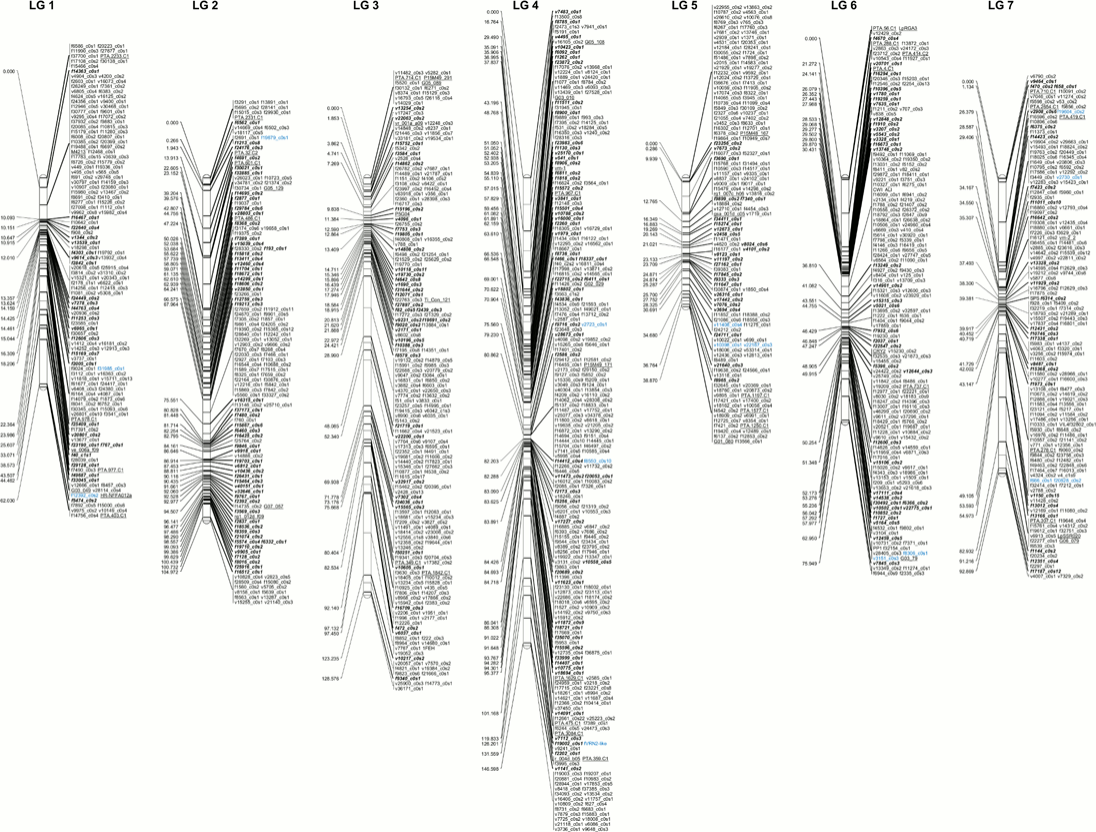
Perennial ryegrass genetic map consisting of candidate genes for vernalization and cold response. High confidence F2-type map presenting the F2 type markers (bold italics) based on which the QTL analysis was performed. The remaining markers are presented in bins. Markers underlined are common with the perennial ryegrass genetic map previously developed from a comprehensive Expressed Sequence Tag (EST) collection [31]. Markers presented in blue were positioned on the map based on co-location on the same genomic scaffold with a mapped marker.

The high confidence F2-type map consists of 257 SNPs showing an F2 type segregation pattern, and has a total length of 649.86 cM distributed across the seven LG (Table 1, Fig 1). The order of markers in the F2-type map was confirmed by the mapping function of the *qtl* R package. Setting this map as a fixed order, the remaining SNPs were added and placed at their most plausible positions (Fig 1, detailed in S5 File). Using a perennial ryegrass draft genome assembly, fifteen additional candidate genes were positioned on the map based on co-location on the same genomic scaffold with a mapped marker. The final map comprises 1,232 markers, out of which 1,175 correspond to transcripts showing expression level changes during vernalization treatment and the shift to long day conditions. The remaining 57 markers are part of the perennial ryegrass genetic map previously developed based on a wide range of marker types [31], and the presence of these markers allows the alignment of the two maps. The use of the ML algorithm enabled us to place the candidate gene based markers into their most plausible position (S5 File).

**Table 1:** Details of the SNP-based linkage map. The F2-type map was built using only the SNPs which showed F2 type segregation. The final map includes all available SNPs, along with 57 previously mapped markers [31].

Linkage group	Length (cM)	No. of markers			
		F2-type markers	Candidate gene-derived markers	Previously mapped markers	Total no. of markers
1	62.030	25	146	8	154
2	104.972	54	130	7	137
3	128.576	39	187	8	195
4	146.598	55	243	11	254
5	38.870	24	141	7	148
6	75.949	36	170	8	178
7	92.869	24	158	8	166
**TOTAL**	**649.864**	**257**	**1,175**	**57**	**1,232**

**Fig 2:**
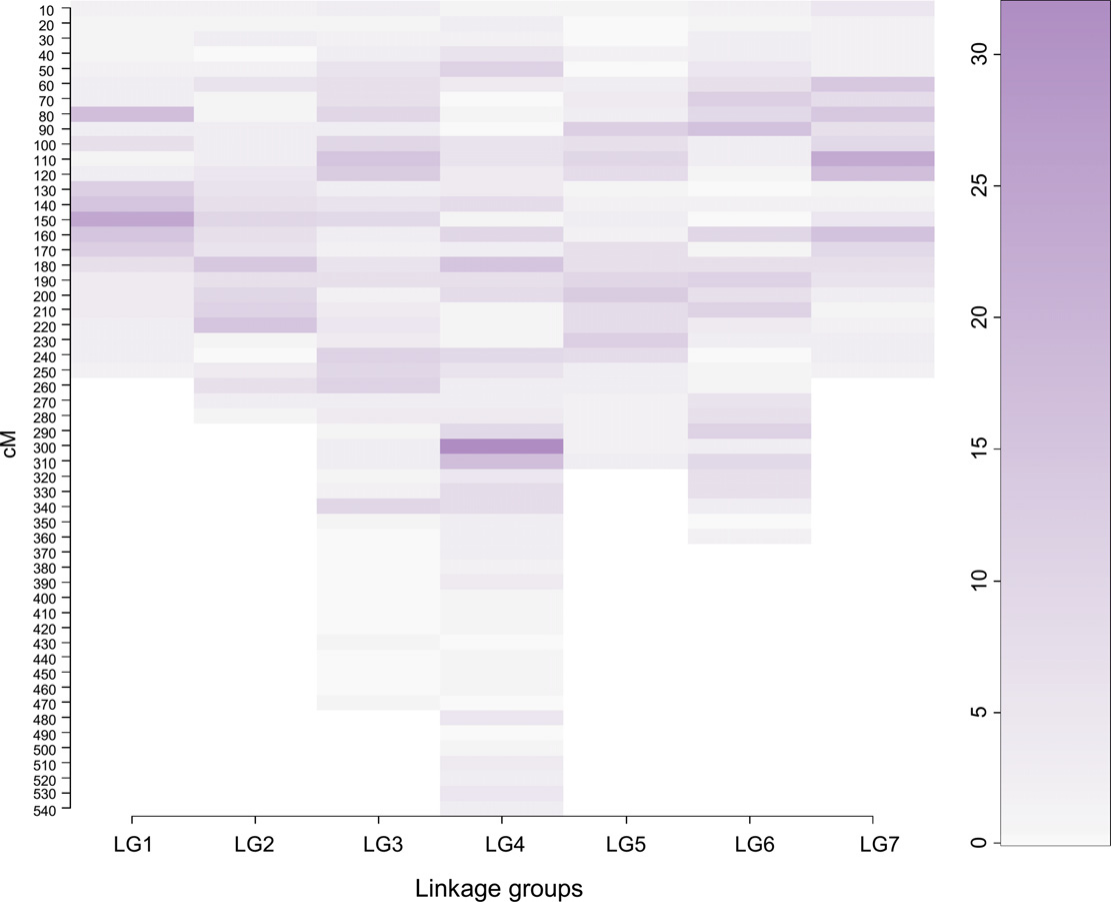
Marker density in the candidate gene-based genetic map. The linkage groups are presented on the *x* axis. The *y* axis corresponds to the most plausible positions in cM on the final candidate gene based genetic map. The color scale illustrates the number of candidate genes mapped in intervals of 10 cM.

In the final map comprising all markers, LG4 had the highest number of candidates, 243, and LG2 had the lowest number, 130. We also observed differences in marker density across the seven LG. All LG had regions with higher and lower marker density, suggestive of genomic regions with clusters of candidate genes for vernalization and cold response (Fig 2). Making direct comparisons between genetic maps constructed using different algorithms is not always straightforward. However, there are clear differences between the clustering pattern of markers on this candidate gene based map and on the transcriptome map previously developed from neutral markers [31].

The SNP discovery pipeline yielded variants with high success rates when transferred to the genotyping array. The high-throughput oligo-pool genotyping array developed for candidates related to vernalization, induction of flowering, and cold responses, together with the SNP marker discovery pipeline, represent a valuable resource for the forage and turf grass community.

The candidate gene based genetic map provides a useful resource for the transfer of information related to map and gene positions across conserved genomic regions of related species. A high level of synteny and macrocolinearity was observed between perennial ryegrass, barley, rice, brachypodium and sorghum, based on which the perennial ryegrass GenomeZipper was developed as a targeted approach towards genome resources for comparative genomics in grass species [40]. Mapping of additional genes in this study augment the GenomeZipper and will improve comparative genomics studies, facilitating cross-species comparative genome analyses of these candidate genes and traits.

Previous studies already highlighted the strong potential of high-throughput SNP genotyping assays for high resolution genetic mapping [41–43], but also for genome wide association studies [44–46], diversity and linkage disequilibrium studies [47, 48]. The assembly of the perennial ryegrass genome was recently published [25] and the presented genetic map can prove useful for anchoring and orienting genome scaffolds, as previously shown for soybean [49] and watermelon [50].

The perennial ryegrass genome is highly repetitive, estimated to be 76% [25, 51]. A total of 590 scaffolds from the perennial ryegrass genome assembly [25] could be anchored using markers combined from both the candidate gene based genetic map and the transcriptome map [31]. This corresponds to over 51.9 Mb, approximately 4.6% of the perennial ryegrass genome, with a density of 12 markers/Mb, covering a total of 1,841 predicted genes on the seven chromosomes, including genes of interest with respect to flowering and cold response. LG5 anchored the lowest number of scaffolds, 20, with 45 predicted genes, while using the markers from LG4 we were able to anchor 150 scaffolds, with 478 predicted genes (Table 2).

**Table 2:** The number of genomic scaffolds anchored to the perennial grass genome assembly.

Chromosome (LG)	No. of scaffolds anchored	No. of genes predicted
1	35	137
2	90	291
3	106	357
4	150	478
5	20	45
6	89	253
7	100	280

The candidate gene based map alone allowed anchoring 470 genome scaffolds, 40.63 Mb and approximately 3.6% of the genome, with 19 of these scaffolds locating two markers.

### Winter survival and heading date

Both winter survival and vernalization response measured as days to heading have been shown to be highly heritable. A recent study based on 1,453 perennial ryegrass F2 families monitored in seven different geographical locations throughout Europe, reported noteworthy heritability values of 0.64 for winter survival and 0.67 for heading date [33]. These values were observed across the F2 families and were location specific. A strong influence of the environment on the WS of plants was indicated by the lower heritability value of 0.16 across locations, a value comparable to across location heritabilities for other traits.

Falster is a Danish perennial ryegrass ecotype adapted to colder winters and has a strong vernalization requirement in order to flower. Veyo is an Italian Mediterranean perennial ryegrass variety without any vernalization requirement in order to flower. Furthermore, the two genotypes showed transcriptome level differences related to genes involved in cold acclimation [26], representing useful experimental material to study vernalization, the induction of flowering, and cold responses.

The phenotypic assessment of WS revealed significant differences between the parental genotypes and between the individuals of the mapping population, indicating that these genotypes represent valuable plant material to study WS (S6 File).

A significant positive correlation (p<0.00002) was found between WS and vernalization response measured as growing degree-days to heading (GDDH) (Table 3). This correlation supports the observed tendency of genotypes with early heading and no requirement for vernalization to have a lower WS, while genotypes with strong vernalization requirement and later heading show on average a better WS.

**Table 3:** Spearman’s rank correlation coefficient for the traits winter survival (WS) and heading date expressed as growing degree-days to heading (GDDH 2004 and GDDH 2005).

Trait	WS	GDDH 2004	GDDH 2005
**GDDH 2004**	0.3860773 (p = 6.767e-06)		
**GDDH 2005**		0.802383 (p<2.2e-16)	
**WS**			0.3677918 (p = 1.946e-05)

WS QTL were detected on LG 2, 3, 4, and 6, with LOD scores between 3.655 and 5.577, and explained variances between 6.91% and 10.92% (Table 4). An interaction was detected between the second QTL on LG4 and the QTL on LG6 (p<0.01) with a LOD score of 3.267 and accounting for 6.13% of the variance.

**Table 4:** QTL for heading date expressed as growing degree-days to heading (GDDH) and for winter survival (WS). QTL signals below the genome wide 3.18 LOD value threshold of GDDH 2005 are presented in italics.

Trait	LG	LOD score	Position (cM)	Closest F2-type marker	1.5 LOD support interval (cM)	95% Bayesian credible interval (cM)	Variation explained
**GDDH 2004**	**2**	**3.73**	**99.629**	**f7128_c0s2**^+^	**47.22–104.97**	**48–104**	**3.74%**
	**3**^p^	**6.55**	**20.813**	**v9231_c0s2**^+^**, f19691_c0s2**^+^	**15.34–24.42**	**15.89–23**	**6.86%**
	**3**	**4.29**	**58**	**v22200_c0s1**	**40–66**	**43–64**	**4.34%**
	**4**^p^	**22.57**	**57.015**	**f15572_c0s2**^+^	**55.11–59.45**	**56–58**	**30.19%**
	**6**^p^	**3.75**	**26.079**	**f10396_c0s5**^+^	**0–69**	**0–68**	**3.76%**
	**7**^p^	**13.30**	**38.3**	**v11929_c0s2**^+^	**35.3–47**	**36–46**	**15.42%**
**GDDH 2005**	*1*	*2*.*82*	*10*.*093*	*f16467_c0s1*^+^	*0–62*.*03*	*0–62*.*03*	*3*.*04%*
	**2**	**5.69**	**53.684**	**f193_c0s1**^+^	**48–61**	**48–66**	**6.4%**
	**3**^p^	**4.12**	**19**	**f2439_c0s3**	**12–41**	**12–41**	**4.53%**
	**4**^p^	**16.24**	**52**	**v25170_c0s1**	**47–57**	**50–56**	**21.4%**
	*6*	*2*.*89*	*21*.*272*	*v20701_c0s1*^+^	*3–75*.*94*	*13–75*.*94*	*3*.*12%*
	**7**^p^	**16.02**	**41.729**	**v8487_c0s1**^+^	**36–47**	**37.49–45**	**16.02%**
**WS**	**2**	**5.19**	**62.939**	**f18606_c0s2**^+^	**59.07–70**	**57.74–70**	**10.09%**
	**3**	**3.66**	**93**	**f16709_c0s3**	**83–110**	**84–109**	**6.91%**
	**4**	**5.58**	**48.768**	**f8900_c0s1**^+^	**44–68**	**46–65**	**10.92%**
	**4**	**5.48**	**139**	**f2202_c0s1**	**124–146.59**	**57–146.59**	**10.7%**
	**6**	**5.58**	**26.352**	**v1780_c0s1**^+^	**14–60**	**2–68**	**10.92%**
	4*6	**3.27**	139*26.35				*6.13%

* Interaction between the second QTL on LG4 and the QTL on LG6 (p < 0.01).

^+^ marker located at the QTL signal peak.

^p^ previously published QTL (Studer et al., 2008).

The WS QTL on LG2 covered 44 transcripts (S5 File). The confidence interval of this QTL overlapped nine markers within the confidence interval of the GDDH QTL detected on this LG only in 2005, the year in which plants were exposed to a longer winter (S7 File).

One of the WS QTL on LG4 located close to the GDDH QTL. A total of 37 transcripts mapped within its confidence intervals (S5 File). The second WS QTL on LG4 covered eight transcripts, including a heat shock protein, a proline rich protein, *S-norcoclaurine synthase 1*-like, and a *flavonol sulfotransferase*-like sequence.

The WS QTL on LG6 located close to the GDDH QTL (Table 4). In case of the WS QTL on LG 2, 3 and 4, the Falster alleles increased WS of the plants, while on LG6 the Falster alleles showed the opposite effect (S1 Fig).

The WS QTL on LG2, 4 and 6 combined explain 48.76% of the total phenotypic variation. Each QTL individually accounted for between 10 and 10.92% of the variance. The smaller effect QTL present on LG3 accounted for 6.91% of the phenotypic variance, with its confidence interval covering 28 transcripts.

We re-ran the QTL analysis for heading date using the new map and previously published phenotype data [11]. The results were consistent with the QTL for heading date previously identified in this population. The largest effect QTL were located on LG4 and LG7, with the QTL on LG4 explaining the highest amount of the variance for heading date. This QTL explained 21.40%–30.19% of the phenotypic variance, and spanned 23 candidate genes (S5 File). The GDDH QTL on LG7 had the second largest effect, explaining 15.42–16.02% of the phenotypic variance, covering a total of 93 transcripts (S5 File).

GDDH QTL were previously identified using the VrnA mapping population [10, 11]. These studies however, were based mostly on anonymous markers. Using SNP markers developed from candidate genes involved in vernalization, induction of flowering, and cold response related processes and basing the analysis on fully informative markers, we were able to establish a set of genes as candidates for these QTL. The two major effect GDDH QTL on LG4 and LG7 were located in the regions previously reported in the VrnA population, but also in populations with different genetic background and different geographic locations [8, 13].

The lack of common markers makes it difficult to relate previously reported WS QTL to the ones detected in the present study. However, the coincident QTL on LG4 for GDDH and WS lie in a similar position to the previously mapped coincident QTL for frost tolerance and heading date in wheat [52–54]. This is based on synteny between the wheat and barley homeologous group 5 chromosomes harbouring these QTL and the ryegrass LG4 [24, 55, 56]. In the same region, WS and freezing tolerance QTL were reported in perennial ryegrass and meadow fescue [24]. This WS QTL overlaps with the GDDH QTL (Table 4). The *VRN1* gene marked on the map by the *vrn-1* marker (Fig 1, S5 File), has previously been proposed to underlie the major effect heading date and vernalization response QTL on perennial ryegrass LG4 [10, 16]. The *vrn-1* marker locate in the GDDH QTL region. The corresponding transcript showed increasing levels throughout vernalization and into the long days in both Falster and Veyo leaves [26]. *VRN1* was described as being connected to both vernalization response and freezing tolerance QTL in barley, and was also shown to exhibit significant associations with both these traits [4, 57–60]. As previously suggested, this evidence points towards potential pleiotropic effects of the *VRN1* gene. In total, there were 23 candidate genes mapped within the confidence interval of the GDDH QTL on LG4, including the *VRN1* gene (S5 File), and the expression profiles of these transcripts are suggestive of involvement in processes related to vernalization and induction of flowering [26]. The possibility of several genes on this LG having a strong effect on vernalization requirement, as observed in meadow fescue [61], cannot be excluded. It is also possible that genes within this region are under some common transcriptional regulation. This has already been observed at the *FLOWERING LOCUS C* (*FLC)* locus in arabidopsis, where genes proximal to *FLC* were repressed in response to vernalization in a manner similar to *FLC*, due to decreased histone H3 acetylation [62].

Another potentially coincident QTL for WS and GDDH was observed on LG6. In spite of the low effects of the GDDH 2005 QTL on this LG and the large confidence interval, the close location of the WS and GDDH signal peaks suggest possible pleiotropic effects in this region, but with opposite effects compared to the region on LG4 (S1 Fig).

#### QTL locate in regions where clusters of candidate genes have been mapped

The candidate genes were distributed across the genome, however, there were clear regions where candidate genes occurred in clusters (Fig 2). The largest cluster was observed on LG4, where 32 candidates were placed within 10 cM. The second largest cluster was observed on LG1, where 24 candidates were placed within 10 cM. Interestingly, a candidate gene with homology to the barley *CONSTANS 9* (*CO9)*-like repressor of flowering (*v6965_c0s1*) mapped in this region. *HvCO9* was described in the photoperiod flowering pathway in barley as a repressor of flowering under short days [63]. The putative *CO9*-like orthologue transcript decreased in abundance with the drop in temperature under short days in both Falster and Veyo genotypes, suggesting its involvement in cold response and/or induction of flowering related processes in perennial ryegrass. No significant QTL were detected in this region. However, a putative GDDH QTL was detected in 2005, the year with a longer winter (S7 File), that fell just short of the threshold determined by permutation (Table 4).

The third largest cluster was observed on LG7, where 23 transcripts mapped within a distance of 10 cM, and this cluster was coincident with a heading date QTL. Furthermore, a number of genes that have previously been shown to play a role in the transition to flowering are also located at this cluster. The GDDH QTL on LG7 covers the marker previously mapped at the signal peak of this QTL using the VrnA population, *vrn2_2* [11, 16]. The alignment of this map with the previously developed perennial ryegrass transcriptome map, locates the GDDH QTL signal peak only a few cM below the *LpCO* and *LpFT* genes, previously proposed as candidates for the LG7 heading date QTL [8, 16]. Furthermore, both *LpCO* and *LpFT* showed significant association with heading date in LD-based association studies [64, 65]. A *trehalose-6-phosphate synthase* (*TPS*)-like sequence (*f12431_c0s2*) was located in the same QTL region. *TPS1* catalyzes the synthesis of threhalose-6-phosphate, a signaling molecule for sugar availability in plants [66], and it was recently shown to play a crucial role in the transition to flowering in *Arabidopsis thaliana* [67]. The presence of *TPS1* was proved essential for flowering also in the presence of promoting environmental conditions, with flowering being very much delayed in *A*. *thaliana* plants lacking *TPS1*. In leaves, *AtTPS1* was shown to be involved in the induction of the *FT* gene that in turn acts to promote the transition to flowering. In the shoot apical meristem, *TPS1* was shown to be involved in the regulation of components of the age dependent flowering pathway. The *TPS*-like transcript, mapped at the perennial ryegrass LG7 heading date QTL, showed down-regulation at the end of vernalization only in Falster leaves. This profile observed for the genotype with vernalization requirement could suggest a signal that the plant is not yet ready to flower at the end of vernalization and before the secondary induction takes place. Among other candidates mapped in this region is a *cinnamoyl CoA reductase*-like sequence (*f12888_c0s1*). This gene was mapped to the barley chromosome 7H, and was found to be significantly associated with heading date in a genome wide association study [60].

All the three QTL detected on LG2 are positioned in regions with clusters of candidate genes. Two candidates of interest covered by GDDH and WS QTL correspond to the *ICE RECRYSTALIZATION INHIBITION protein* transcript (*f193_c0s1*) and *CONSTITUTIVE PHOTOPORPHOGENESIS 10* protein transcript (*v22850_c0s1*). A winter survival QTL with highest significance was reported on meadow fescue LG2, 20 cM distal to the *PHOTOPERIOD 1* (*FpPPD1*) gene [24]. The WS QTL detected in the present study in perennial ryegrass mapped 40 cM distal to *LpPPD1* and could correspond to the same meadow fescue QTL.

The proximal GDDH QTL on LG3 as well as the GDDH and WS QTL on LG6 overlap high candidate gene density regions. Surprisingly, none of the QTL detected on LG4 localized in a high density region. The second WS QTL on LG4 covered genes typically described as having a role in cold and stress responses. Heat shock proteins and heat shock transcription factors are at the convergence of multiple stress response pathways, including cold stress [68], while proline accumulates in response to cold [69] conferring freezing tolerance and functions as a cryoprotectant in the cells [70]. Norcoclaurine synthase and flavonol sulfotransferase were related to plant defence and stress responses [71, 72]. The *VERNALIZATION 2*-like (*VRN2*-like) gene, a repressor of flowering in monocots down-regulated by vernalization and short days [73–75], was positioned on the genetic map based on co-location on the same genomic scaffold with the norcoclaurine synthase-like marker. A recent study described a different expression profile for the *Brachypodium dystachyon VRN2*-like gene compared to wheat and barley, with increasing levels under vernalization [76].

Genome wide association studies in perennial ryegrass are likely to be based on a candidate gene approach in the near future. This is because linkage disequilibrium decays very rapidly in perennial ryegrass [65, 77–79], meaning full genome re-sequencing of the association panel is the most likely methodology to succeed in an untargeted approach. The differentially regulated candidate genes clustering in regions overlapping with QTL, identified here, make ideal targets for candidate gene based association analysis for winter survival and heading date in perennial ryegrass.

## Conclusions

We have developed a candidate gene based genetic map that places a total of 1,175 candidate genes for cold acclimation and induction of flowering on the perennial ryegrass genetic map. We used the map to identify QTL for winter survival and relate them to previously identified QTL for heading date. A positive correlation was observed between strong vernalization requirement and winter survival, and QTL for winter surivival and heading date overlapped in two regions on the genetic map. Candidate genes were located in clusters along the genetic map, some of which co-localized with QTL for winter survival and heading date. These clusters of candidate genes may be used in candidate gene based association studies to identify alleles associated with winter survival and heading date.

## Supporting Information

S1 Fig Genotype distributions for markers located at the signal peak or closest to the peak of the QTL identified for heading date (GDDH 2004, GDDH 2005) and for winter survival (WS) in the VrnA mapping population. Markers positioned on the same linkage group (LG) are placed in the same row. The blue bars correspond to genotypes homozygous for the allele originating from the Falster genotype, symbolized ‘AA’ on the *x* axis. The red bars correspond to genotypes homozygous for the Veyo allele., symbolized ‘BB’ on the *x* axis. The purple bars corresponds to heterozygous genotypes symbolized ‘AB’ on the *x* axis. The *y* axis illustrates the phenotypic scores for heading date expressed as growing degree-days to heading recorded in the years 2004 and 2005 (GDDH 2004, GDDH 2005) and for winter survival (WS). The position on the F2-type map is indicated for each marker. *marker co-localizing with the QTL signal peak.
(PDF)

S1 File SNP identification steps. (PDF)

S2 File SNP validation results using High Resolution Melting. (XLSX)

S3 File Sequences including the SNP markers as present on the Illumina GoldenGate genotyping array. The SNP positions are marked within the sequence as [variant/variant].
(TXT)

S4 File ALLMAPS chain file presenting the anchored scaffolds. (TXT)

S5 File Candidate gene based genetic map of perennial ryegrass. (XLSX)

S6 File Assessment of winter survival of the VrnA population. (PDF)

S7 File Average temperature recorded during the phenotyping period for heading date expressed as growing degree-days to heading of the VrnA population. (PDF)
