# Supplementary figures and images for: Using a Candidate Gene-Based Genetic Linkage Map to Identify QTL for Winter Survival in Perennial Ryegrass
Source: PLoS One. 2016 Mar 24;11(3):e0152004. doi: 10.1371/journal.pone.0152004 (PMC4807000; doi:10.1371/journal.pone.0152004)

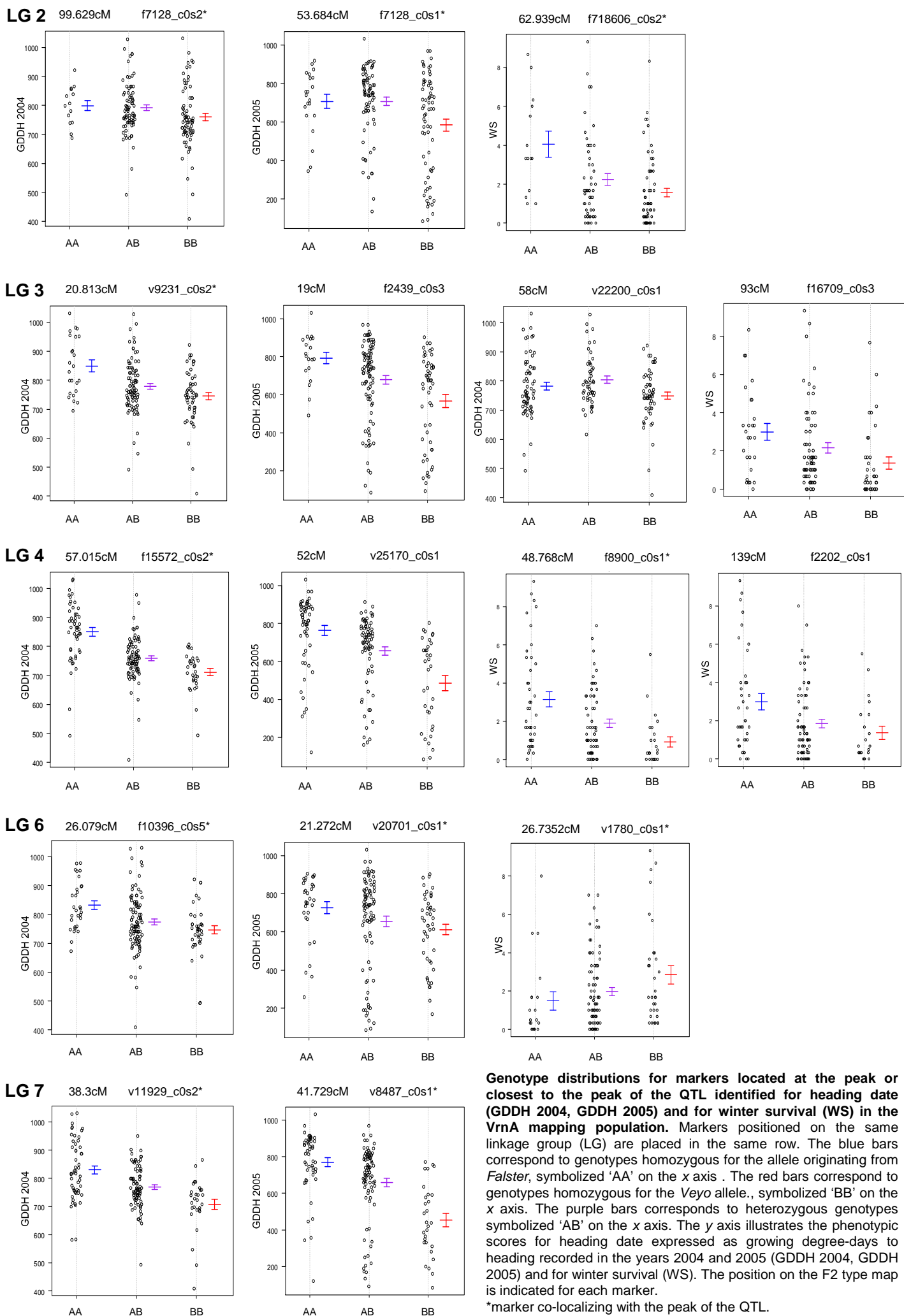

Supplement: S1 Fig — Markers positioned on the same linkage group (LG) are placed in the same row. The blue bars correspond to genotypes homozygous for the allele originating from the Falster genotype, symbolized ‘AA’ on the x axis. The red bars correspond to genotypes homozygous for the Veyo allele., symbolized ‘BB’ on the x axis. The purple bars corresponds to heterozygous genotypes symbolized ‘AB’ on the x axis. The y axis illustrates the phenotypic scores for heading date expressed as growing degree-days to heading recorded in the years 2004 and 2005 (GDDH 2004, GDDH 2005) and for winter survival (WS). The position on the F2-type map is indicated for each marker. *marker co-localizing with the QTL signal peak. (PDF) [file pone.0152004.s001.pdf]
