# Supplementary material for: Using a Candidate Gene-Based Genetic Linkage Map to Identify QTL for Winter Survival in Perennial Ryegrass
Source: PLoS One. 2016 Mar 24;11(3):e0152004. doi: 10.1371/journal.pone.0152004 (PMC4807000; doi:10.1371/journal.pone.0152004)
Supplement: S1 File — (PDF) [file pone.0152004.s002.pdf]

SNP identification steps

The number of SNPs after each applied filter, as well as the percentage related to the initial number of SNPs are presented separate for the two genotypes, Falster and Veyo.

Filter no.	Filter description	No. of SNPs		% of SNPs
		Falster	Veyo	(Falster / Veyo)
1	Select for 5x coverage and 3 reads supporting the variant	23,967	21,097	100%
2	Keep only biallelic SNPs	23,964	21,075	99.98% / 99.89%
3	Select for SNPs with a variant frequency between 10 – 90%	16,492	14,683	68.81% / 69.59%
4	Select for no other SNPs within 60 bp flanking sequence	10,278	9,157	42.88% / 43.40%
5	Select for variant frequency between 25 – 75%	6,597	6,026	27.52% / 28.56%
6	Eliminate SNPs failed by the strand filter	6,403	5,873	26.71% / 27.83%
7	Select SNPs in transcripts which hit the genome	6,384	5,792	26.63% / 27.45%
8	Select for no indels/introns within 60 bp flanking sequence	3,175	2,810	13.24% / 13.32%
9	Eliminate mitochondrial and chloroplastic sequences	3,175	2,810	13.24% / 13.32%
	Total	5,985		

	Falster	Veyo	Total
Total number of transcript sequences with SNPs	1,385	1,109	2,494
