# Supplementary material for: Using a Candidate Gene-Based Genetic Linkage Map to Identify QTL for Winter Survival in Perennial Ryegrass
Source: PLoS One. 2016 Mar 24;11(3):e0152004. doi: 10.1371/journal.pone.0152004 (PMC4807000; doi:10.1371/journal.pone.0152004)
Supplement: S6 File — (PDF) [file pone.0152004.s007.pdf]

Assessment of winter survival of the VrnA population

Phenotypic trait distribution for the trait winter survival. The parental phenotypes and population mean are indicated with arrows.

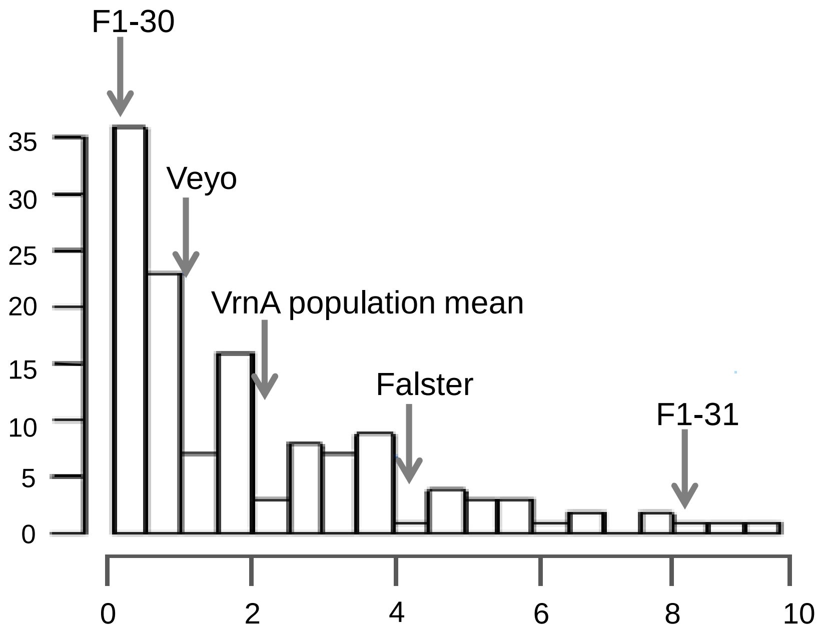

Analysis of variance of winter survival scores based on comparison of the mean scores of the VrnA family within replicates with the scores of the grandparents (GP=Falster and Veyo) and the parents (P=F1-30 and F1-39). The differences between the mean scores of the VrnA family, GP and P are tested using orthogonal contrasts.

Source	Df	MS	F-value	P
Replication	2	0.5639	0.72	0.5153
Genotype	4	28.9204	36.98	<0.0001
VrnA vs GP and P	1	4.0982	5.24	0.0513
GP vs P	1	6.7500	8.63	0.0188
Between GP	1	88.1667	112.73	<0.0001
Between P	1	16.6667	21.31	0.0017
Residual	8	0.7821		
