# Supplementary material for: Using a Candidate Gene-Based Genetic Linkage Map to Identify QTL for Winter Survival in Perennial Ryegrass
Source: PLoS One. 2016 Mar 24;11(3):e0152004. doi: 10.1371/journal.pone.0152004 (PMC4807000; doi:10.1371/journal.pone.0152004)
Supplement: S7 File — (PDF) [file pone.0152004.s008.pdf]

Average temperature recorded during the phenotyping period for heading date expressed as growing degree-days to heading of the VrnA population

	Mean of daily maximum temperature (^oC)		Mean of daily minimum temperature (^oC)		Mean temperature (^oC)	
Month	year of phenotyping		year of phenotyping		year of phenotyping	
	2004	2005	2004	2005	2004	2005
February	15.895	9.72	4.463	4.592	10.179	7.156
March	14.548	16.316	5.096	5.533	9.822	10.925
April	23.407	22.7	8.392	7.064	15.9	14.882
May	22.121	25.166	8.857	11.466	15.489	18.31
June	21.153		11.506		16.33	
